# Examining the Role of Race in Maternal Morbidities and Adverse Infant Birth Outcomes in Florida (2004 to 2022) – A Retrospective Exploratory Analysis

**DOI:** 10.1007/s40615-025-02599-z

**Published:** 2025-08-13

**Authors:** Alexa Parra, Raymond Balise, Vanessa Morales, Cynthia N. Lebron, Yue Pan, JoNell Potter, Hudson P. Santos

**Affiliations:** 1 School of Nursing and Health Studies, University of Miami, 5030 Brunson Dr, Coral Gables, FL 33146 USA; 2 Miller School of Medicine, Department of Public Health, University of Miami, 1120 NW 14Th St, Miami, FL 33136 USA

**Keywords:** Maternal and infant health, Maternal and child health, Black hispanic, Afro-latina, Hispanic, Maternal morbidity, Adverse infant outcomes

## Abstract

**Background:**

Studies reporting maternal morbidities and adverse infant outcomes group Hispanics together without considering the implications of racial identity, particularly among Black Hispanics in the United States.

**Objective:**

This study aims to examine maternal morbidity and adverse infant birth outcomes across racially and ethnically diverse groups in Florida (from 2004–2022), with particular attention to the heterogeneity within the Hispanic population.

**Methods:**

We conducted a retrospective secondary analysis of Florida birth certificate data from 2004 to 2022 (N = 3,364,973), examining maternal morbidity (hypertensive disorders in pregnancy, gestational diabetes) and adverse infant outcomes (preterm birth, small or large for gestational age). Outcomes were stratified by race and ethnicity. Logistic regression models were employed to estimate crude and adjusted odds ratios while controlling for key sociodemographic covariates.

**Results:**

Black Hispanic mothers had higher odds of hypertensive disorders in pregnancy, but no significant difference in gestational diabetes after considering the independent impact of race and ethnicity. Their infants had lower odds of preterm birth and small-for-gestational-age, and higher odds of large-for-gestational-age after considering the independent impact of race and ethnicity. Compared to other groups, Black Hispanics had the lowest rates of private insurance, educational attainment, and prenatal care adequacy, highlighting sociodemographic disparities alongside clinical outcomes.

**Conclusion:**

Our findings highlight nuanced disparities in maternal and infant outcomes for Black Hispanics. Further investigation is essential to understand why maternal health outcomes for Black Hispanics align more closely with their Black counterparts, while infant outcomes diverge, emphasizing the need for disaggregated analyses within the Hispanic population.

## Introduction

Maternal morbidity, including hypertensive disorders in pregnancy and gestational diabetes, and adverse infant birth outcomes, such as preterm birth and abnormal birth weight, remain major public health concerns in the United States (U.S.) [1]. These complications contribute to long-term health risks for both pregnant mothers and their infants, including higher rates of chronic diseases, infant mortality, and long-term developmental issues[2]. Studies have demonstrated that Black non-Hispanic mothers are disproportionately at a higher risk for maternal morbidities[3, 4]. Black non-Hispanic mothers are three times more likely to die due to pregnancy-related causes when compared to White non-Hispanic mothers [5]. In contrast, the “Hispanic/Latina epidemiological paradox” indicates that Hispanic mothers have birth outcomes comparable to or better than non-Hispanic White mothers, with lower rates of low birthweight and infant mortality[6]. However, there is some evidence that Hispanic mothers may have slightly higher odds for low birth weight [6]compared to White non-Hispanic mothers. These conflicting findings underscore that homogenizing the Hispanic population is not a sufficient approach to actionable health research[7]. The “Hispanic/Latina epidemiological paradox” conceptualizes the Hispanic population as a monolith and may lead to the oversight of critical lived experiences that have the potential to influence health outcomes[8].

Hispanic maternal and child health (MCH) outcomes vary greatly when considering mothers’ immigration status, country of origin, acculturation, and socioeconomic status [6, 9]. For example, the Hispanic Community Health Study/Study of Latinos (HCHS/SOL) found that mothers of Mexican, Puerto Rican, or Dominican descent had higher prevalence of self-reported gestational diabetes than mothers of Central American, Cuban, or South American descent [10]. Similarly, higher acculturation levels are associated with prematurity, low birthweight, teen pregnancy, and neonatal mortality[11]. The heterogeneity of the Hispanic population is further demonstrated by varying racial identities. In the U.S. Black Hispanics^1^ (commonly referred to interchangeably in the literature as Afro-Latinas, Afro-Hispanics, Afro-Caribbeans, or Black Latina/o [12–14]) may be disadvantaged in health outcomes due to the intersectionality of multiple minority identities[8, 13, 15] (being racially Black, ethnically Hispanic, and women) compared to their other Hispanic counterparts. The inconsistent nomenclature used to describe this population in the literature highlights the need for standardization to better characterize the unique MCH health outcomes associated with their experiences[16, 17].

The lived experiences of Afro Latinas are shaped by race, skin tone, and other factors in ways that differ from other Hispanics (e.g., White Hispanics) but are similar to Black non-Hispanic individuals, such as experiencing discrimination[12]. The burden of health disparities may be more pervasive among Afro-Latinas because they may experience distinctive stressors of societal disadvantages based on race and skin color[18–20]. Although skin color is not recorded in vital statistics data, Afro Latinas may share some of the phenotypic features of Black non-Hispanics, including skin color, associated with discrimination, racism, Social Determinants of Health (SDoH), and structural inequities[18, 19, 21, 22].

Our prior work, including a systematic review, highlighted the scarcity of research into MCH outcomes for Afro-Latinas [9, 16]. Notably, the extent to which being racially Black is a risk factor for these maternal morbidities and infant birth outcomes among Afro-Latinas is poorly understood. Determining the prevalence of these adverse perinatal and infant outcomes among Afro Latinas is essential in preventing long-term complications, including more extended hospital stays, neonatal intensive care admission, future maternal diabetes, and infant mortality[10, 18, 20, 23, 24]. Given the heterogeneity of the Hispanic population, and the disparities in MCH outcomes that racial and ethnic minority groups face, additional inquiry is needed to adequately meet the health needs of these distinct populations.

This study aims to examine maternal morbidity and adverse infant birth outcomes across racially and ethnically diverse groups, with particular attention to the racial heterogeneity within the Hispanic population. Traditional MCH research often limits comparisons to broad categories such as Black race, White race, and Hispanic ethnicity, overlooking important within-group differences. By disaggregating Hispanic ethnicity to include Black Hispanics and Other Hispanics, this study challenges these conventional groupings and seeks to determine whether Black Hispanics and their infants experience outcomes more similar to their Black (traditional categorization) counterparts. We hypothesize that adverse outcomes among Black Hispanics will more closely mirror those of Black populations, highlighting the need for greater nuance in demographic classifications used in MCH research to advance health equity.

## Methodology

This study was a retrospective, secondary, exploratory analysis of maternal morbidity (i.e., hypertensive disorders in pregnancy and gestational diabetes) and adverse infant birth outcomes (i.e., preterm birth and small or large for gestational age) among mothers^2^ and their infants in Florida, stratified by mothers’ self-identified race and ethnicity.

### Study Population and Data Sources

This study used birth certificate (vital statistics) data from the Florida Department of Health (FL DOH). Florida is the fourth most populous U.S. state, with 21.5 million people; it comprises 67 counties and is further subdivided into 11,442 census block groups[25]. Among the states with the highest Hispanic population, Florida is third behind California and Texas[25]. Compared to the national average of 18%, Florida has one of the largest Hispanic populations in the U.S (26%) [26]. The average racial composition of Florida is 53.2% White non-Hispanic, 16.9% Black non-Hispanic, 26.5% Hispanic, and 3.0% Asian[25].

In the U.S., state laws require birth certificates to be completed for all births, and federal law mandates national collection and publication of birth and other vital statistics data[27]. The National Vital Statistics System, the Federal compilation of this data, is the result of the cooperation between the National Center for Health Statistics (NCHS) and states to provide access to statistical information from birth certificates[27]. Standard forms for collecting data and model procedures for the uniform registration of the events are developed and recommended for state use through cooperative activities of the states and NCHS [27].

Upon request and complying with the FL DOH data use agreements, birth certificate data were received for 4,009,855 live births for infants born in Florida from 2004 to 2022. This database contains extensive information on every documented live birth in Florida (2004–2022), including but not limited to maternal age, birth weight, clinical estimate of gestation, plurality, maternal complications, congenital anomalies, maternal tobacco use, and a variety of maternal and paternal demographic variables. Access to the data and methods for receiving, storing, linking, and analyzing data and presenting results is governed by a research protocol approved by the FL DOH’s Institutional Review Board (IRB).

**Fig. 1:**
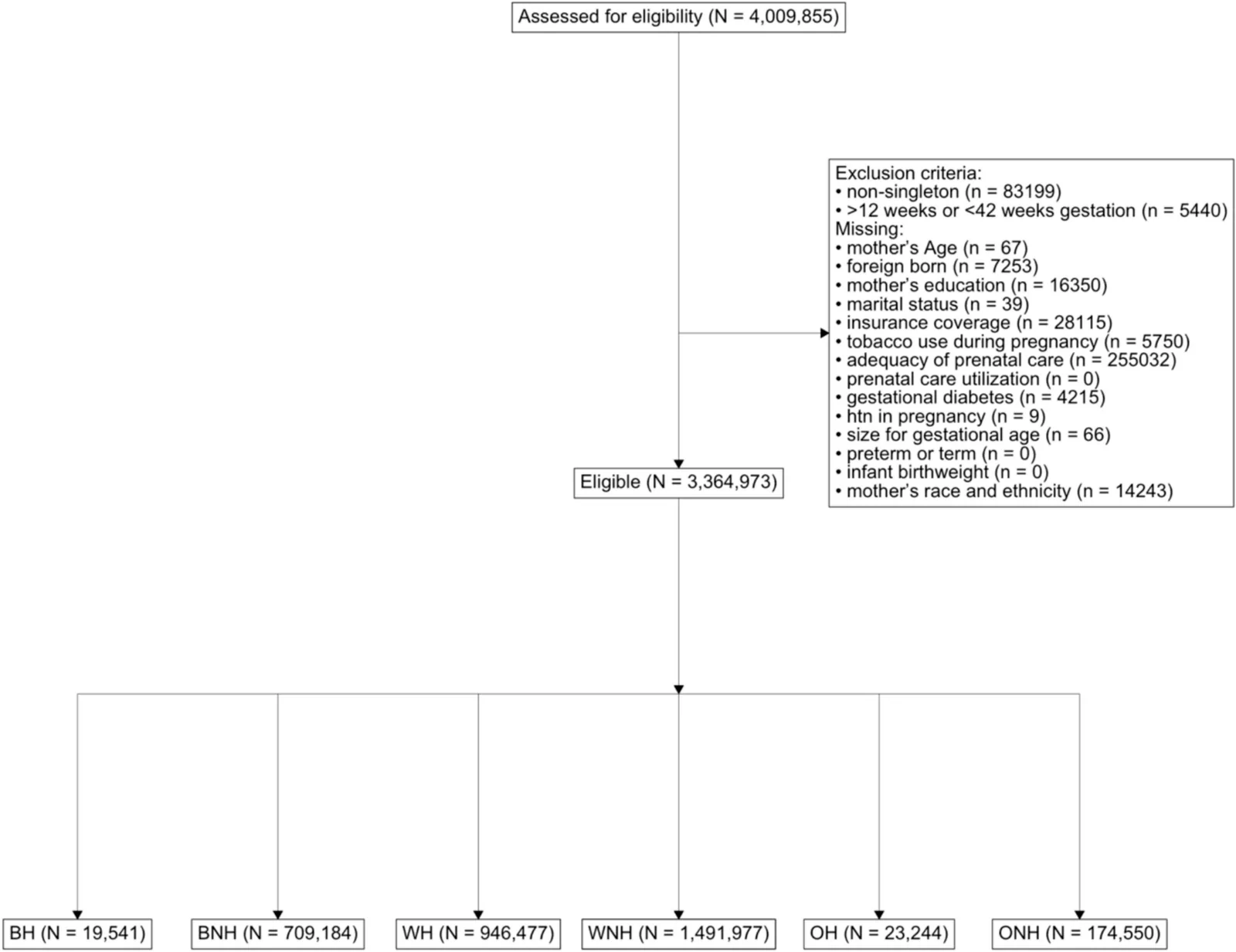
Consort Diagram. *BH* Black Hispanics, *BNH* Black non-Hispanics, *WH* White Hispanics, *WNH* White non-Hispanics, *OH* Other Hispanics, *ONH* Other non-Hispanics

Variables extracted from this data set included, but were not limited to, birth outcome, sex of infant, maternal characteristics, and demographics such as self-reported race, ethnicity, age, marital status, education, medical disease during pregnancy, prenatal care initiation, and type of insurance coverage. Eligible participants selected for this study were: (1) singleton live births (stillbirths and neonatal death data are not available in this dataset) and (2) gestational age between 22 and 42 weeks at delivery. Mothers or infants missing variables of interest (e.g., maternal race, ethnicity, gestational age, infant weight) were excluded from the analysis (Fig. 1**)*.*** The final analysis dataset included a total of 3,364,973 live births.

### Measures

#### Maternal Morbidity Outcomes

Maternal morbidity, i.e., medical disease during pregnancy, was extracted from the original dataset, which included hypertensive disorders in pregnancy (gestational hypertension, preeclampsia, eclampsia) and pregnancy-related diabetes (gestational diabetes). Hypertensive Disorders in Pregnancy and Gestational Diabetes were selected based on the existing ( MCH literature; pregnancy-related hypertension is established as a prevalent perinatal morbidity and risk factor for serious health complications and mortality[18, 24, 28]. Further, Black non-Hispanic and Hispanic mothers have higher rates of gestational diabetes[29]. These variables were coded as categorical (yes, no, or unknown). Gestational hypertension is defined in pregnant people as a recorded systolic and diastolic blood pressure greater than or equal to 140 and/or 90 mm Hg [19]. Severe-range hypertension is blood pressure greater than or equal to 160/110 mm Hg[30]. Hypertensive Disorders in Pregnancy in the dataset (and our analysis) include chronic hypertension, gestational hypertension, preeclampsia, and chronic hypertension with superimposed preeclampsia. Gestational diabetes is defined as diabetes mellitus developed for the first time during pregnancy[31]. All categories were coded as yes, no, or unknown.

#### Infant Birth Outcomes

Adverse infant birth outcomes include preterm delivery and abnormal size for gestational age (excluding any infant with a weight within normal limits). Size for gestational age was categorized using the birth weight (in grams) and weeks of gestation at birth using the Fenton Growth Chart [32]. Size for gestational age was classified as (1) small for gestational age (SGA) and (2) large for gestational age (LGA). An infant is determined to be SGA if the newborn weighed < 10th percentile or LGA if > 90th percentile of the birth weight distribution for a given gestational week, based on the infant’s sex[33]. Preterm birth is defined as gestational age < 37 weeks based on an obstetric estimate of gestational age. Less than > 22 weeks were not included in this analysis, as those pregnancies are not considered viable, typically resulting in neonatal death [34–36]. Preterm birth for the study sample was determined using weeks of gestation on the day of birth. All categories were coded as yes, no, or unknown.

#### Sociodemographic Factors

Age, marital status, insurance type, educational level, prenatal tobacco use, and prenatal care utilization were collected from the Florida birth certificate data. Maternal characteristics were categorized by age: ≤ 19 as teenage pregnancy, 20–24 as young adult pregnancy, and ≥ 35 as advanced maternal age. The Adequacy of Prenatal Care Utilization (APNCU) Index [37], also called the Kotelchuck Index, is a tool used to classify the number of prenatal visits compared to the expected number of visits for the period between when care began and the delivery date. The proportion of observed visits/expected visits is calculated, and results are scaled into four categorical groups: inadequate, intermediate, adequate, and adequate plus. The expected number of visits is based on the American College of Obstetricians and Gynecologists’ prenatal care standards [38]. It is adjusted for the gestational age when care began and the gestational age at delivery [37]. The APNCU/Kotelchuck index is already determined in the provided dataset using categorical variables (inadequate, intermediate, adequate, and adequate plus)[37].

#### Maternal Race and Ethnicity

The self-identified race recorded on the birth certificate was defined as Black, White, or Other. The self-identified ethnicity was described as Hispanics, Not Hispanics, or Other. These categories were pre-defined in the birth certificate data as self-selected by the birthing people recorded. All categories were coded as yes, no, or unknown.

Two categorization approaches were used to reflect both conventional demographic classifications and intersectional identities relevant to this study. For the descriptive analysis (Table 1), race and ethnicity were combined into six mutually exclusive categories: Black Hispanics (BH), Black non-Hispanics (BNH), White Hispanics (WH), White non-Hispanics (WNH), Other Hispanics (OH), and Other non-Hispanics (ONH). This grouping structure allowed for a comprehensive sociodemographic and clinical profile of the study population.

**Table 1:** Demographic Characteristics by Racial/Ethnic Group

	BH	BNH	WH	WNH	OH	ONH	
	(N = 19,541)	(N = 70,9184)	(N = 946,477)	(N = 1,491,977)	(N = 23,244)	(N = 174,550)	P-value
**Age at Delivery**							
Mean (SD)	27.2 (6.04)	27.0 (6.21)	28.3 (6.10)	28.7 (5.82)	27.8 (6.15)	29.9 (5.69)	< 0.001
Median [Min, Max]	27.0 [12.0, 51.0]	26.0 [11.0, 57.0]	28.0 [11.0, 61.0]	29.0 [11.0, 62.0]	28.0 [11.0, 49.0]	30.0 [12.0, 56.0]	
**Marital Status**							
No or Widowed	12,314 (63.0%)	485,991 (68.5%)	465,685 (49.2%)	504,589 (33.8%)	11,843 (51.0%)	43,975 (25.2%)	< 0.001
Yes	7,227 (37.0%)	223,193 (31.5%)	480,792 (50.8%)	987,388 (66.2%)	11,401 (49.0%)	130,575 (74.8%)	
**Insurance coverage**							
Medicaid	11,863 (60.7%)	472,412 (66.6%)	452,690 (47.8%)	550,672 (36.9%)	11,955 (51.4%)	54,534 (31.2%)	< 0.001
Self-pay	1,069 (5.5%)	38,628 (5.4%)	146,943 (15.5%)	56,715 (3.8%)	2,448 (10.5%)	15,090 (8.6%)	
Private Insurance	6,134 (31.4%)	181,907 (25.7%)	318,412 (33.6%)	838,572 (56.2%)	7,550 (32.5%)	97,663 (56.0%)	
Other	475 (2.4%)	16,237 (2.3%)	28,432 (3.0%)	46,018 (3.1%)	1,291 (5.6%)	7,263 (4.2%)	
**Educational Attainment**							
12 grade or less	2,629 (13.5%)	123,698 (17.4%)	220,803 (23.3%)	130,435 (8.7%)	5,128 (22.1%)	14,400 (8.2%)	< 0.001
High School or GED	6,978 (35.7%)	289,594 (40.8%)	303,711 (32.1%)	397,265 (26.6%)	6,717 (28.9%)	39,086 (22.4%)	
Some college	4,720 (24.2%)	147,694 (20.8%)	145,725 (15.4%)	293,280 (19.7%)	4,618 (19.9%)	27,036 (15.5%)	
Bachelors or Associates degree	4,383 (22.4%)	120,371 (17.0%)	220,135 (23.3%)	506,708 (34.0%)	5,409 (23.3%)	64,444 (36.9%)	
Graduate or Doctoral degree	831 (4.3%)	27,827 (3.9%)	56,103 (5.9%)	164,289 (11.0%)	1,372 (5.9%)	29,584 (16.9%)	
**Mother Foreign Born**							
No	11,539 (59.1%)	527,978 (74.4%)	320,964 (33.9%)	1,363,137 (91.4%)	11,060 (47.6%)	54,078 (31.0%)	< 0.001
Yes	8,002 (40.9%)	181,206 (25.6%)	625,513 (66.1%)	128,840 (8.6%)	12,184 (52.4%)	120,472 (69.0%)	
**Tobacco use in Pregnancy**							
No/Former	19,103 (97.8%)	689,238 (97.2%)	935,643 (98.9%)	1,356,739 (90.9%)	22,636 (97.4%)	170,238 (97.5%)	< 0.001
Yes	438 (2.2%)	19,946 (2.8%)	10,834 (1.1%)	135,238 (9.1%)	608 (2.6%)	4,312 (2.5%)	
**Prenatal Care**							
No	330 (1.7%)	19,359 (2.7%)	14,102 (1.5%)	15,803 (1.1%)	421 (1.8%)	2,662 (1.5%)	< 0.001
Yes	19,211 (98.3%)	689,825 (97.3%)	932,375 (98.5%)	1,476,174 (98.9%)	22,823 (98.2%)	171,888 (98.5%)	
**Adequacy of Prenatal Care**							
Inadequate	3,137 (16.1%)	147,183 (20.8%)	148,836 (15.7%)	175,995 (11.8%)	4,880 (21.0%)	26,862 (15.4%)	< 0.001
Intermediate	2,904 (14.9%)	108,074 (15.2%)	143,666 (15.2%)	207,147 (13.9%)	3,376 (14.5%)	26,691 (15.3%)	
Adequate	8,328 (42.6%)	264,154 (37.2%)	382,903 (40.5%)	680,518 (45.6%)	9,173 (39.5%)	75,247 (43.1%)	
Adequate Plus	5172 (26.5%)	189,773 (26.8%)	271,072 (28.6%)	428,317 (28.7%)	5815 (25.0%)	45,750 (26.2%)	
**Preterm Birth**							
No (Term)	17,868 (91.4%)	627,963 (88.5%)	876,007 (92.6%)	1,385,591 (92.9%)	106,386 (7.1%)	161,386 (92.5%)	< 0.001
Yes	1,673 (8.6%)	81,221 (11.5%)	70,470 (7.4%)	106,386 (7.1%)	1,804 (7.8%)	13,164 (7.5%)	
**Gestational Diabetes**							
No	18,622 (95.3%)	677,392 (95.5%)	897,861 (94.9%)	1,421,521 (95.3%)	21,986 (94.6%)	159,764 (91.5%)	< 0.001
Yes	919 (4.7%)	31,792 (4.5%)	48,616 (5.1%)	70,456 (4.7%)	1,258 (5.4%)	14,786 (8.5%)	
**Hypertension in Pregnancy**							
No	18,330 (93.8%)	660,882 (93.2%)	903,137 (95.4%)	1,405,308 (94.2%)	22,069 (94.9%)	167,244 (95.8%)	< 0.001
Yes	1,211 (6.2%)	48,302 (6.8%)	43,340 (4.6%)	86,669 (5.8%)	1,175 (5.1%)	7,306 (4.2%)	
**Mother's age by Category**							
Teenagers	1,806 (9.2%)	76,609 (10.8%)	70,155 (7.4%)	83,152 (5.6%)	2,028 (8.7%)	6,679 (3.8%)	< 0.001
Young adults	15,089 (77.2%)	536,396 (75.6%)	715,740 (75.6%)	1,156,206 (77.5%)	17,585 (75.7%)	129,921 (74.4%)	
Advanced maternal age	2,646 (13.5%)	96,179 (13.6%)	160,582 (17.0%)	252,619 (16.9%)	3,631 (15.6%)	37,950 (21.7%)	
**Size by Gestational Age**							
SGA	2,207 (11.3%)	101,839 (14.4%)	79,504 (8.4%)	107,739 (7.2%)	2,207 (9.5%)	20,373 (11.7%)	< 0.001
Average	15,874 (81.2%)	565,409 (79.7%)	783,398 (82.8%)	1,221,955 (81.9%)	19,103 (82.2%)	142,367 (81.6%)	
LGA	1,460 (7.5%)	41,936 (5.9%)	83,575 (8.8%)	162,283 (10.9%)	1,934 (8.3%)	11,810 (6.8%)	

*BH* Black Hispanics, *BNH* Black non-Hispanics *WH* White Hispanics, *WNH* White non-Hispanics, *OH* Other Hispanics, *ONH* Other non-Hispanics

For regression analyses, maternal race and ethnicity were reclassified into five analytic groups: Black race (independent of ethnicity), Other race (independent of ethnicity), Hispanic ethnicity (independent of race), White non-Hispanics (reference group), Black Hispanics, and Other Hispanics. This categorization strategy was employed to align with traditional demographic constructs commonly used in MCH research while accounting for intersectionality. Specifically, the Black Hispanics interaction and Other Hispanics interaction were included to assess whether intersecting racial and ethnic identities revealed disparities not captured by broader race or ethnicity categories alone.

### Statistical Analysis

Sociodemographic, maternal, and infant characteristics were summarized and reported using descriptive statistics (See Table 1). Categorical variables were expressed as frequencies and percentages. Pearson Chi-square (χ^2^) tests were used to evaluate group differences across the six mutually exclusive race/ethnicity categories in the descriptive analysis (Table 1).

Univariate and multivariate logistic regression analyses examined the relationship between race and ethnicity and maternal and infant outcomes (Tables 2 and 3). White non-Hispanics served as the reference group for all comparisons. The regression models included both traditional and intersectional groupings to evaluate the independent effects of Black race and Hispanic ethnicity and the interaction effects observed among Black Hispanics and Other Hispanics mothers and their infants.

**Table 2:** Logistic Regression Results for Differences in Maternal Morbidity^*^ for Race and Ethnicity

	Base Model				Multivariate Model^a^		
	OR	[95% CI]	*p*		OR	[95% CI]	*p*
**Gestational Diabetes**							
**Race/Ethnicity**^**b**^							
White Non-Hispanic	REF	-	-		REF	-	-
Hispanic^†^	1.09	[1.08, 1.1]	<.001		1.07	[1.05, 1.08]	0.617
Black^‡^	- 0.95	[0.93, 0.96]	<.001		- 1.0	[0.98, 1.01]	<.001
Black Hispanic	- 0.96	[1.9, 1.03]	0.272		- 0.99	[0.93, 1.06]	0.843
Other Race^†††^	1.87	[1.83, 1.9]	<.001		1.78	[1.75, 1.81]	<.001
Other Hispanic	- 0.57	[0.53, 0.6]	<.001		- 0.62	[0.58, 0.65]	<.001
**Hypertensive Disorders in Pregnancy**							
**Race/Ethnicity**^**b**^							
White Non-Hispanic	REF	-	-		REF	-	-
Hispanic^†^	- 0.78	[0.77, 0.79]	<.001		- 0.79	[0.78, 0.8]	<.001
Black^‡^	1.19	[1.17, 1.2]	<.001		1.15	[1.14, 1.17]	<.001
Black Hispanic	1.16	[1.09, 1.23]	<.001		1.15	[1.09, 1.23]	<.001
Other Race^†††^	- 0.71	[0.69, 0.73]	<.001		- 0.73	[0.71, 0.74]	<.001
Other Hispanic	1.57	[1.47, 1.67]	<.001		1.53	[1.44, 1.63]	<.001

^*^ Gestational Diabetes is diabetes mellitus developed for the first time during pregnancy. Hypertensive Disorders in Pregnancy include chronic hypertension, gestational hypertension, preeclampsia, and chronic hypertension with superimposed preeclampsia

^†^ Hispanic (independent of race)

^‡^ Black (independent of ethnicity)

^†††^ Other race (independent of ethnicity)

^a^Included covariates: maternal education, maternal age, marital status, prenatal care utilization (prenatal care utilization and Kotelchuck Index), type of insurance coverage (Medicaid, private, self-pay, other), smoker (during pregnancy), and infant sex as a biological variable (ref: male)

^b^Impact (main effect) for race and ethnicity and their interaction (i.e., Black*Hispanic and Other*Hispanic), controlling for all other predictors

**Table 3:** Logistic Regression Results for Differences in Infant Birth Outcomes^*^ for Race and Ethnicity

	Base Model				Multivariate Model^a^		
	OR	[95% CI]	*p*		OR	[95% CI]	*p*
**Preterm Birth**							
**Race/Ethnicity**							
White Non-Hispanic	REF	-	-		REF	-	-
Hispanic^†^	1.05	[1.04, 1.06]	<.001		1	[1, 1.02]	0.344
Black^‡^	1.69	[1.67, 1.7]	<.001		1.57	[1.55, 1.58]	<.001
Black Hispanic	- 0.69	[0.66, 0.73]	<.001		- 0.76	[0.72, 0.8]	<.001
Other Race^†††^	1.06	[1.04, 1.08]	<.001		1.12	[1.1, 1.15]	<.001
Other Hispanic	- 0.98	[0.93, 1.04]	0.559		- 0.96	[0.91, 1.01]	0.127
**SGA**							
**Race/Ethnicity**							
White Non-Hispanic	REF	-	-		REF	-	-
Hispanic^†^	1.18	[1.17, 1.19]	<.001		1.19	[1.18, 1.21]	<.001
Black^‡^	2.15	[2.13, 2.17]	<.001		2.02	[2, 2.04]	<.001
Black Hispanic	- 0.64	[0.62, 0.68]	<.001		- 0.66	[0.63, 0.69]	<.001
Other Race^†††^	1.7	[1.67, 1.73]	<.001		1.88	[1.85, 1.91]	<.001
Other Hispanic	- 0.67	[0.64, 0.71]	<.001		- 0.59	[0.56, 0.62]	<.001
**LGA**							
**Race/Ethnicity**							
White Non-Hispanic	REF	-	-		REF	-	-
Hispanic^†^	- 0.79	[0.79, 0.8]	<.001		- 0.78	[0.77, 0.78]	<.001
Black^‡^	- 0.52	[0.51, 0.52]	<.001		- 0.54	[0.53, 0.55]	<.001
Black Hispanic	1.62	[1.53, 1.71]	<.001		1.63	[1.54, 1.72]	<.001
Other Race^†††^	- 0.6	[0.58, 0.61]	<.001		- 0.56	[0.55, 0.57]	<.001
Other Hispanic	1.58	[1.5, 1.66]	<.001		1.73	[1.65, 1.82]	<.001

^*^Infant birth outcomes – Preterm birth is defined as gestational age < 37 weeks based on the obstetric estimate of gestation, SGA if the newborn weighed < 10th percentile, and LGA if > 90 percentile of the birth weight distribution for a given gestational week, based on the infant’s sex

^†^Hispanic (independent of race)

^‡^Black (independent of ethnicity)

^†††^ Other race (independent of ethnicity

^a^Included covariates: maternal education, maternal age, marital status, prenatal care utilization (prenatal care utilization and Kotelchuck Index), type of insurance coverage (Medicaid, private, self-pay, other), smoker (during pregnancy), and infant sex as a biological variable (ref: male)

^b^Impact (main effect) for race and ethnicity and their interaction (i.e., Black*Hispanic and Other*Hispanic), controlling for all other predictors

In the multivariate logistic regression models, sociodemographic factors of interest (maternal education, age, marital status, prenatal care, principal payment source for delivery, and tobacco use during pregnancy) were controlled, with two-tailed hypothesis tests considering statistical significance set at p < 0.05 for all analyses. Crude and adjusted odds ratios (OR) with 95% CI were presented. All statistical analyses were performed using R software (version 4.3.2) with the conflicted(1.2.0)[39], supernova (3.0.0)[40], Table 1 (1.4.3)[41], broom (1.0.6)[42], forestplot(3.1.3)[43], ggconsort (0.1.0)[44], labelled (2.13.00)[45], and tidyverse (2.0.0)[46] packages.

## Results

Statistically significant differences were observed across multiple maternal and infant characteristics (see Table 1). The study sample consisted of six racial and ethnic groups, with the largest representation among White non-Hispanics (reference group) (N = 1,491,977), followed by White Hispanics (N = 946,477), Other non-Hispanics (N = 174,550), Black non-Hispanics (N = 70,9184), Other Hispanics (N = 23,244), and the smallest group being Black Hispanics (N = 19,541). The mean age at delivery ranged from 27.0 years (SD = 6.21) to 29.9 years (SD = 5.69). Marital status (indicating being married) showed notable variation, with the largest share of married mothers found among White Non-Hispanics (66.2%), followed by Other non-Hispanics (75.2%) and White Hispanics (50.8%), while the lowest percentage was among Black Hispanics (37.0%).

Insurance coverage also differed notably; the highest proportion of private insurance users was among White non-Hispanics (56.2%), followed by Other non-Hispanics (56.0%) and White Hispanics (33.6%), whereas the lowest percentage was observed among Black Hispanics (31.4%). Educational attainment displayed clear disparities, with White non-Hispanics holding the highest percentage of bachelor’s degrees or higher at 45.0%, followed by other non-Hispanic mothers at 39.1%, and Black Hispanics had the lowest rate at just 7.9%. Foreign-born status was most prevalent among White Hispanics (66.1%) and Black non-Hispanics (25.6%), while White non-Hispanics had the lowest percentage (8.6%).

Prenatal care utilization was high across all groups, with the highest proportion receiving adequate-plus care among White non-Hispanics (28.7%), followed by White Hispanics (28.6%), and the lowest among Black Hispanics (26.5%). Tobacco use during pregnancy was most frequent among White non-Hispanic (9.1%), followed by Other non-Hispanics (2.5%), with the lowest prevalence among Black Hispanics (2.2%).

### Maternal Morbidities

#### Gestational Diabetes

In the base model, Black Hispanic mothers had lower odds of developing gestational diabetes compared to White non-Hispanics (reference group) (Table 2), with an OR of 0.96 (*p* = 0.272). In contrast, Hispanic mothers and Other-race mothers exhibited significantly higher odds of developing gestational diabetes (OR = 1.09, *p* < 0.001; OR = 1.87, *p* < 0.001). The multivariate model (Table 2), adjusting for covariates such as maternal age, education, marital status, prenatal care, insurance type, smoking status, and infant sex, showed an aOR of 0.99 (* p* = 0.843) for Black Hispanics, suggesting no significant difference compared to White non-Hispanics after controlling for these covariates.

#### Hypertensive Disorders in Pregnancy

The base model (Table 2) demonstrated that Black Hispanics had 16% higher odds of hypertensive disorders in pregnancy (OR = 1.16, *p* < 0.001). Black mothers and Other Hispanic mothers also had significantly higher odds, 19% and 57% higher (OR = 1.19, *p* < 0.001; OR = 1.57, *p* < 0.001). Conversely, Hispanics had 22% lower odds, and Other-race mothers had 29% lower odds (OR = 0.78, *p* < 0.001; OR = 0.71, *p* < 0.001).

These disparities persisted in the multivariate model (Table 2). Black Hispanics and Black mothers both had 15% higher odds (aOR = 1.15, *p* < 0.001; aOR = 1.15, *p* < 0.001). Other Hispanic mothers had 53% higher odds (aOR = 1.53, *p* < 0.001). In contrast, Hispanics and Other-race mothers had 22% lower odds and 29% lower odds, respectively (aOR = 0.78, *p* < 0.001; aOR = 0.71, *p* < 0.001).

### Adverse Infant Birth Outcomes

#### Preterm Birth

Infants born to Black Hispanics had 31% lower odds of preterm birth (OR = 0.69,* p* < 0.001), a finding that persisted after adjustment for covariates (aOR = 0.76, *p* < 0.001). In contrast, infants born to Black mothers had 69% higher odds of preterm birth in the base model (OR = 1.69, *p* < 0.001), and 57% higher odds in the adjusted model (aOR = 1.57, *p* < 0.001). Infants of Other-race mothers also had significantly higher odds of preterm birth, 6% higher in the base model (OR = 1.06, *p* < 0.001) and 12% higher after adjustment (aOR = 1.12, *p* < 0.001).

#### Small-for-Gestational-Age (SGA)

Infants of Black Hispanic mothers had significantly lower odds of being SGA compared to those of White non-Hispanics (see Table 3), and this remained significant after adjustment, with a 34% lower odds (aOR = 0.66, *p* < 0.001). Similarly, infants of Other Hispanic mothers had 33% lower odds of SGA in the base model (OR = 0.67, *p* < 0.001) and 41% lower odds after adjustment (aOR = 0.59, *p* < 0.001).

On the other hand, infants of Black mothers had more than twice the odds of SGA (See Table 3), which remained significantly elevated after adjustment (aOR = 2.02, *p* < 0.001). Infants of Other-race mothers also had increased odds, 70% higher in the base model (See Table 3) and 88% higher in the adjusted model (aOR = 1.88, *p* < 0.001).

#### Large-for-Gestational-Age (LGA)

Black Hispanic infants had 62% higher odds of being LGA compared to the reference group in the base model (see Table 3), which persisted in the adjusted model (aOR = 1.63, *p* < 0.001). Similarly, infants of Other Hispanic mothers had 58% higher odds of LGA in the base model (OR = 1.58, *p* < 0.001) and 73% higher odds in the adjusted model (aOR = 1.73,* p* < 0.001).

Conversely, infants of Black mothers had lower odds of LGA (see Table 3), which remained consistent and statistically significant in the multivariate model with 46% lower odds (aOR = 0.54, *p* < 0.001). Infants of Other-race mothers had 40% lower odds (OR = 0.60, *p* < 0.001), and 44% lower odds after adjustment (aOR = 0.56, *p* < 0.001). Infants of Hispanics also had significantly lower odds of LGA, 21% in the base model (OR = 0.79, *p* < 0.001) and 22% after adjustment (aOR = 0.78, *p* < 0.001).

## Discussion

According to the limited literature available[11, 38–40], we hypothesized that outcomes for Black Hispanic mothers and their infants would resemble those of Black mothers and infants (traditional categorization). This hypothesis was supported for hypertensive disorders in pregnancy but not for gestational diabetes or the adverse infant outcomes. Infants of Black mothers had elevated odds of preterm birth and SGA. In contrast, Black Hispanics had lower odds of these outcomes, suggesting divergent patterns in risk.

These findings contribute to a more nuanced understanding of MCH disparities by highlighting the distinct experiences of Black Hispanics, an often-overlooked population in research [17]. While our analysis did not reveal universally elevated risks among Black Hispanic maternal morbidities and adverse infant outcomes, the observed patterns underscore the importance of examining outcomes within specific racial and ethnic subgroups, rather than aggregating populations.

One of the most striking findings was the contrasting pattern of infant birth outcomes between Black Hispanics and Black mothers. For nearly every infant outcome examined, these two groups exhibited opposing trends. These disparities suggest that shared racial identity does not equate to shared health risks, particularly when modified by ethnicity and potentially other intersecting factors such as nativity, acculturation, or structural exposure [8, 15, 15, 17]. Further investigation is warranted to explore the unique risk profiles of Black Hispanics and Other Hispanics, and to identify both protective factors and disparities that may arise from their intersectional identities.

Additionally, we found that Black Hispanics had the lowest rates of private insurance, educational attainment, and prenatal care adequacy, highlighting sociodemographic disparities alongside clinical outcomes. These patterns highlight sociodemographic disadvantages that may contribute to or exacerbate disparities in these perinatal outcomes, even when clinical risks are not uniformly elevated. This underscores the importance of examining clinical indicators and the broader context of structural and SDoH. Future research should explore how factors such as income, neighborhood deprivation, immigration status, and access to prenatal care shape perinatal outcomes among Black Hispanics. Building on our findings in this study, our next study will investigate the role of SDoH in influencing maternal morbidity and adverse birth outcomes within this population to identify targeted strategies to reduce inequities.

### Implications

The results of our analysis underscore the complexity of racial and ethnic disparities in MCH, particularly for populations at the intersection of multiple identities. We know that for a majority of MCH research and interventions, Black and Hispanic identities have been treated as mutually exclusive categories. The population that identifies as both requires additional studies to assess and address disparities [4] accurately, [6, 42, 43]. Given the heterogeneity within the Hispanic population, these findings reinforce the importance of examining MCH outcomes through a more granular lens that considers both racial and ethnic identities. Although the magnitude of disparities in some outcomes was modest for Black Hispanics, their distinct profile, lower socioeconomic indicators, lower prenatal care adequacy, and unique risk patterns signal the need for targeted research and interventions. Current MCH literature often conflates diverse Hispanic subgroups, which may obscure disparities faced by Afro-Latinas, a group whose dual identities subject them to overlapping systems of disadvantage.

Understanding how race and ethnicity intersect to influence birth outcomes for Black Hispanics is relevant to the current discourse on racial disparities and perinatal inequities in the U.S. Additionally, by examining perinatal outcomes based on comprehensive maternal racial and ethnic identification, we can develop hypotheses about the root causes of these disparities, highlight the implications of changing demographics in the U.S., and offer guidance for designing future interventions tailored to the diverse population of Hispanic mothers and infants [40].

### Strengths and Limitations:

A key strength of this study is its large-scale retrospective cohort design featuring a robust sample size of over 3.3 million live births. By examining trends in maternal characteristics, maternal morbidity, and adverse infant outcomes among Black Hispanic mothers compared to other racial and ethnic groups, this research addresses a critical gap in the limited literature on MCH outcomes for this population in the U.S., particularly in Florida.

However, the study has notable limitations. The reliance on birth certificate data limits the analysis due to the absence of detailed information on socioeconomic status, health behaviors, and other risk factors contributing to disparities. Evidence suggests that the validity of certain MCH indicators on the birth certificate is suboptimal compared to hospital discharge records. For example, one study found that birth certificates substantially underreport maternal morbidity and may underestimate racial disparities when used in isolation[47]. This issue is compounded by findings from another study suggesting that agreement between medical records and birth certificate data varies widely across data elements, while some variables, such as cesarean delivery and birthweight, are reliable, others, like prenatal visits and prior preterm birth, show low agreement[48]. Taken together, while vital statistics provide a valuable foundation for population-level analysis of MCH outcomes, these limitations call for cautious interpretation of findings related to maternal morbidity and adverse infant outcomes. Future studies should consider linking birth certificate data with hospital discharge or electronic health records to enhance validity of health indicators and improve racial disparity estimates'accuracy. Additionally, the APNCU index does not consider the quality of care. It relies on accurate recall by patients or healthcare providers regarding the timing and frequency of prenatal visits.

Another limitation of this study is the potential influence of demographic and contextual changes over the 18-year study period (2004–2022). Florida’s population has become increasingly diverse over time[49]. For example, the proportion of the population identifying as Hispanic/Latino increased from 22.6% in 2010 to 27.1% in 2022, while the share of White non-Hispanic residents declined from 58% to 52.3% during the same period[49]. These shifts may have influenced birth outcomes and access to prenatal care across racial and ethnic groups. While our models adjusted for individual-level covariates, we did not account for population-level changes or examine trends across time. Future studies should consider stratifying outcomes by time period to explore whether demographic transitions or policy changes have differentially impacted MCH among racial and ethnic subgroups.

Further, the complexity of racial and ethnic classification in U.S. vital records. In the U.S., the racial term "Black" has historically been equated with African American individuals, complicating the self-identification process for Hispanics of African descent within the Black racial category [17]. This nuance could impact the representation of Black Hispanic mothers and infants in the study sample, despite its large size, and should be taken into account when interpreting the findings. Future research should consider alternative classification methods, such as incorporating self-identified racial subcategories or phenotype-based assessments, to improve accuracy.

## Conclusion

Overall, our study highlights the importance of disaggregating race and ethnicity, especially among Hispanics, to better understand the unique maternal and infant risks faced by this population in Florida. Our findings indicate that Black Hispanics exhibit maternal morbidity patterns similar to those of Black mothers, such as a higher risk of hypertensive disorders in pregnancy. However, their infants did not show similar patterns in adverse infant outcomes, underscoring the complexity of how race and ethnicity influence these outcomes. These insights stress the necessity for future research to investigate the underlying mechanisms driving these disparities, including structural and SDoH.

Given the growing Black Hispanic population in the U.S., MCH research should shift away from broad racial and ethnic categories that overlook the diversity within groups to develop targeted interventions that can effectively address the unique health challenges faced by this population. Future studies ought to incorporate more comprehensive measures of racial identity, perceived discrimination, and other aspects of healthcare access to guide policies and interventions aimed at reducing racial disparities in maternal and infant birth outcomes.

## Data Availability

Availability of review materials are available upon request.

## Footnotes

1 Throughout this manuscript, we will use the terms"Afro-Latina"and"Black Hispanic"interchangeably to refer to individuals of African descent from Latin American or Hispanic countries, depending on the terminology used in the original data sources cited. Although these terms often overlap, they are not interchangeable. However, for consistency with the original sources and simplicity, we will adopt the terminology as it appears in the referenced data.

2 While we acknowledge that"birthing people"is a more inclusive term, the term"mothers"is used throughout this manuscript to align with the terminology of the original data source (Florida birth certificate data), which classifies individuals who have given birth as"mothers.".

## Code availability

Available upon request.
